# Case report: a unique pediatric case of a primary CD8 expressing ALK-1 positive anaplastic large cell lymphoma of skeletal muscle

**DOI:** 10.1186/1746-1596-7-38

**Published:** 2012-04-12

**Authors:** Timo Gaiser, Eva Geissinger, Torsten Schattenberg, Hanns-Peter Scharf, Matthias Dürken, Dietmar Dinter, Andreas Rosenwald, Alexander Marx

**Affiliations:** 1 Institute of Pathology, Medical Faculty Mannheim, University of Heidelberg, Theodor-Kutzer-Ufer 1-3, 68167 Mannheim, Germany; 2 Orthopedic Clinic, Medical Faculty Mannheim, University of Heidelberg, Theodor-Kutzer-Ufer 1-3, 68167 Mannheim, Germany; 3 Department of Pediatrics, Medical Faculty Mannheim, University of Heidelberg, Theodor-Kutzer-Ufer 1-3, 68167 Mannheim, Germany; 4 Radiology and Nuclear Medicine, Medical Faculty Mannheim, University of Heidelberg, Theodor-Kutzer-Ufer 1-3, 68167 Mannheim, Germany; 5 Section of Cancer Genomics, Center for Cancer Research, National Cancer Institute, National Institutes of Health, National Insitutes of Health, 50 South Drive, Bldg. 50, Bethesda, MD 20892, USA; 6 Institute of Pathology, University of Würzburg, Josef-Schneider-Straße 2/Bau E2, 97080 Würzburg, Germany

**Keywords:** ALK-1, Anaplastic large cell lymphoma, CD30, Pediatric lymphoma

## Abstract

Primary involvement of skeletal muscle is a very rare event in ALK-1 positive anaplastic large cell lymphoma (ALCL). We describe a case of a 10-year old boy presenting with a three week history of pain and a palpable firm swelling at the dorsal aspect of the left thigh. Histological examination of the lesion revealed a tumoral and diffuse polymorphic infiltration of the muscle by large lymphoid cells. Tumor cells displayed eccentric, lobulated "horse shoe" or "kidney-shape" nuclei. The cells showed immunohistochemical positivity for CD30, ALK-1, CD2, CD3, CD7, CD8, and Perforin. Fluorescence in situ hybridization analysis revealed a characteristic rearrangement of the ALK-1 gene in 2p23 leading to the diagnosis of ALK-1 positive ALCL. Chemotherapy according to the ALCL-99-NHL-BFM protocol was initiated and resulted in a complete remission after two cycles. This case illustrates the unusual presentation of a pediatric ALCL in soft tissue with a good response to chemotherapy.

## Background

Anaplastic large cell lymphoma (ALCL) was discovered by Stein et al. and characterized by strong expression of the Ki-1 (CD30) antigen [1]. In 1988, ALCL was included in the revised Kiel classification and is nowadays classified as a Non-Hodgkin lymphoma of T-cell origin by the World Health Organization [2,3]. The key moment in understanding the primary biological driver behind ALCL was the discovery of the recurrent t(2;5)(p23;q35) translocation in this lymphoma type [4]. This finding was further elucidated by Morris and colleagues who identified the two genes involved in this translocation: the anaplastic lymphoma kinase (ALK-1) on chromosome 2 and nucleophosmin (NPM1) on chromosome 5 [5]. Subsequently, the NPM-ALK fusion protein was proven to have oncogenic capacity [6]. Among the four main types of pediatric non-Hodgkin lymphoma (Burkitt lymphoma, diffuse large B-cell lymphoma, anaplastic large cell lymphoma and lymphoblastic lymphoma) ALCL has the best prognosis: event-free and overall survival rates were 72% and 88%, respectively, even in advanced-stage diseases [7].

Here, we report a case of a 10-year old boy diagnosed with an ALK-1 positive anaplastic large cell lymphoma of the skeletal muscle. Primary involvement of skeletal muscle by ALCL is extremely rare and so far only four pediatric cases have been reported in the literature [8-11].

## Case presentation

### Initial presentation and management

**Figure 1:**
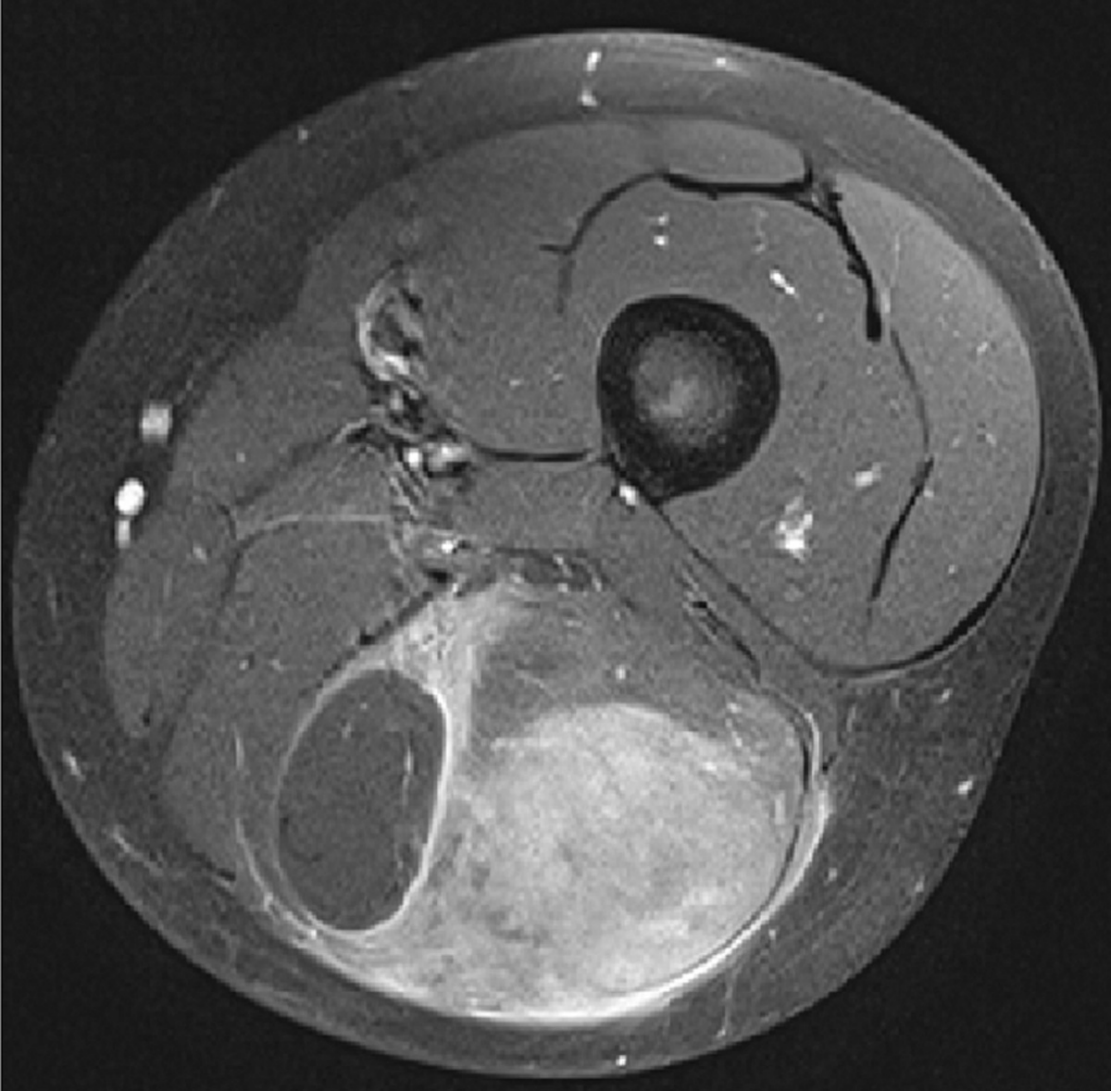
**Axial MRI of the left thigh delineates an ill-defining tumor mass within the long head of the biceps femoris muscle with vivid uptake of contrast media while measuring 4 × 3 × 9 cm in size**.

A 10-year old boy presented with a painful swelling in the dorsal aspect of the left distal thigh without history of trauma. Pain started 3 weeks before, increased with time and resulted in difficulties in walking at time of presentation. No fever, night sweats or weight loss was reported. Physical examination revealed a 9 × 4 cm palpable firm mass in the biceps femoris muscle. Magnetic resonance imaging (MRI) demonstrated a 4 × 3 × 9 cm ill-defining tumor within the long head of the biceps femoris muscle with vivid uptake of contrast medium (Figure 1). Also, enlarged lymph nodes up to a diameter of 2 cm were detected popliteal, inguinal and iliac. Radiologically, the most probable diagnosis was a malignant soft tissue tumor, most likely a rhabdomyosarcoma. Beside slightly enlarged inguinal and popliteal lymph nodes further staging did not reveal other organ or skin involvement. Laboratory HIV 1 + 2 screening tests showed no signs for HIV infection. Histological diagnosis was achieved by incisional biopsy. After being classified as Murphy stage II [12], further treatment was conducted according to the international protocol for childhood ALCL (ALCL-99-NHL-BFM). ALK-1/NPM PCR was found positive in peripheral blood. After two cycles of chemotherapy the tumor was no longer detectable clinically or by ultrasound. The patient is currently receiving further chemotherapy without experiencing major toxicity.

## Materials and methods

### Immunohistochemistry

Four micron tissue sections were stained with the following monoclonal antibodies: CD2, CD7, CD20, CD30, CD31, CD79, CD99, CD117, Alk-1, Desmin, D2-40, EMA, Ki67, TdT (all Dako Cytomation, Glostrup; Denmark), CD3, βF1 (T-cell receptor beta chains) (both Thermo Fisher Scientific, Waltham, MA, USA), CD5, CD8, CD10, CD56, Perforin (Leica, Wetzlar, Germany), and CD34 (Immunotech, Glendale, CA, USA). Antibody binding was visualized using the Envision System as described by the manufacturer.

### In situ hybridization

In situ hybridization analyses for the t(2;5) translocation and for Epstein-Barr virus (EBV) encoded small nuclear RNAs (EBERs) was performed on four micron sections of formalin fixed, paraffin-embedded tissue paraffin sections using the ALK Dual Color, Break Apart Rearrangement Probe (Abbott, North Chicago, IL, USA) and ZytoFast™ EBV Probe (ZytoVision GmbH, Bremerhaven, Germany); procedures were performed according to the manufacturers' indications.

## Results

**Figure 2:**
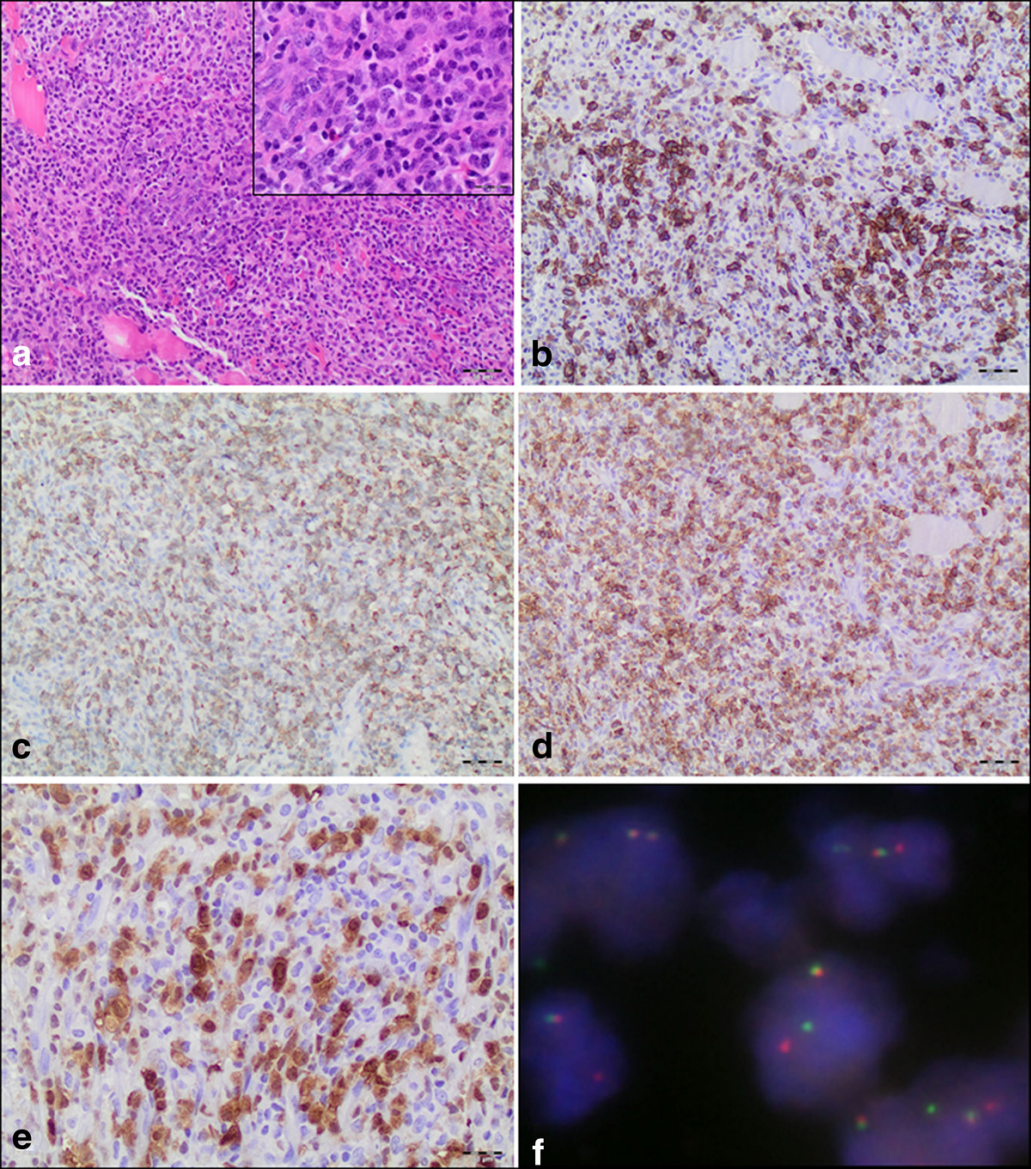
**Histological features and immunophenotype of the intramuscular ALCL**. (A) The biceps femoris muscle showed a tumoral and diffuse polymorphic infiltration by large lymphoid cells (magnification 100×). Inlay: Majority of the cells were small to middle sized with pleomorphic nuclei and various size of pale cytoplasm. Intermingled and less common larger cells with eccentric lobulated "horse shoe" or "kidney-shape" nuclei and an abundant cytoplasm (so called "hallmark cells") (magnification 400×). (B) Strong membranous and perinuclear dot-like positivity for CD30 (magnification 100×). (C) T-cell phenotype of ALCL demonstrated by positivity for CD7 and CD8 (D) (magnification 100×). (E) Tumor cells expressing ALK-1 protein both in the cytoplasm and nucleus indicating the classical t(2;5) translocation (magnification 200×). (F) FISH with ALK Dual Color, Break Apart Rearrangement Probe for detecting translocations affecting 2p23 ALK breakpoint region. ALK locus in its native state is seen as two immediately adjacent red and green signals. However, if a t(2;5) chromosome rearrangement has occurred as in this case, one separate red and one separate green signals can be seen (the one remaining native ALK remains as an orange/green signal) (magnification 1000×).

Histologically, the muscle of the biceps femoris displayed a diffuse polymorphic lymphoid infiltration of high mitotic activity, including atypical mitoses. While no haemangio invasion was demonstrable, lymphovascular invasion was detectable morphologically and by D2-40 immunohistochemistry. The majority of tumor cells was small to middle sized with pleomorphic nuclei and variable amount of pale cytoplasm. Intermingled and less common were larger cells with eccentric lobulated "horse shoe" or "kidney-shape" nuclei and abundant cytoplasm with an intense eosinophilic region representing the Golgi apparatus (Figure 2A). These "hallmark cells" strongly expressed CD30, while the smaller lymphoid cells were only moderately positive (Figure 2B). Furthermore, the tumor cells stained positive for CD2, CD3, CD7 (Figure 2C), CD99, Perforin and, remarkably, CD8 (Figure 2D). ALK-1 had an obvious cytoplasmic and nuclear pattern of immunoreactivity in the tumor cells (Figure 2E). As expected, rearrangement of the ALK-1 gene in 2p23 could be proven by fluorescence in situ hybridization analyses (Figure 2F). The whole tumor cell population revealed a CD5 antigen loss and a weak and obviously only cytoplasmic reactivity for βF1. The Ki67 proliferation index reached ~60%. Tumor cells stained negative for CD10, CD20, CD79a, CD34, CD56, CD117, TdT, Desmin, and EMA. With EBER in situ hybridization no EBV positive cells were detectable. Based on these findings the diagnosis of CD30/ALK-1 positive anaplastic large cell lymphoma was established and confirmed by the reference center for lymphoid malignancies in Würzburg (AR, EG).

## Conclusions

ALCL can be classified into ALK-1 negative and ALK-1 positive ALCL. ALK-1 negative ALCL occurs mainly in older patients (peak incidence ~ 60 y) and in advanced-stage disease. ALK-1 positive ALCL is a clinically aggressive lymphoma that mostly occurs in the first three decades of life [13]. However, overall survival and longer disease free survival is observed after treatment with aggressive chemotherapy. Patients affected by ALK-1 positive ALCL have a significant better overall survival than ALK-1 negative ALCL patients (5-year overall survival: 70-80% vs. 33-49%) [14,15]. It is still controversially discussed if this observation can be explained by the biological role of the ALK-1 fusion proteins or by the younger age of the ALK-1 positive ALCL patients. Unlike ALK-1 positive ALCL, which is characterized by t(2;5)(p23;q35) translocation and resulting in the expression of the NPM-ALK-1 fusion protein, so far no recurrent cytogenetic alterations have been described in ALK-1 negative ALCL.

The presented case illustrates a CD30+, ALK-1+ ALCL of T-cell origin (CD2-/+, CD3+, CD7+, CD8+ and Perforin+) in a pediatric patient. Due to the abundance of small neoplastic cells with pale cytoplasm mixed with some medium-sized and large lymphoid cells this case is best counted among the "small cell variant" of ALCL (10%). Other ALCL subcategories are the common (70-80%) and lymphohistiocytic type (10%).

Primary malignant lymphoma of soft tissue is an infrequent, and often diagnostically challenging neoplasm [16,17]. Among those studies which reported the T- or B-cell phenotype of primary soft tissue lymphoma, B-cell accounted for over 90%. Most of those cases showed aggressive histology and were of diffuse large B-cell phenotype [17]. T cell lymphomas in general and ALCL in particular exceedingly rarely exhibit primary infiltration of skeletal muscle [8-10]. Of note, all intramuscular primary ALCL cases reported so far have been ALK-1 positive. Why this T-cell lymphoma could overcome the normally well established protection of skeletal muscle against lymphocytic infiltration is unknown [18].

Interestingly, the current ALCL is the first among the reported intra-muscular ALCL primaries to show a CD8 phenotype, which is extremely rare in ALCL. However, trauma, which theoretically could have acted as a stimulatory trigger providing signals for T cell homing to skeletal muscle [18] was denied by the patient. Moreover, no other infectious or autoimmune process was known from the history or the local findings.

It should also be mentioned in this context that the slightly enlarged lymph nodes were only detected after MRI examination and were not clinically indicative. Unfortunately a lymph node biopsy was not performed. Therefore, a final statement about a possible secondary involvement of the muscle was not possible. However, we consider this possibility less likely because the majority of ALCL patients (70%) presents with advanced stage III to IV disease [3], which was obviously not the case in our patient.

ALCL has to be distinguished from classical Hodgkin lymphoma (cHL), CD30+ non-Hodgkin B cell lymphomas and very rare ALK-1 positive (and eventually CD30-negative) large B cell lymphomas. Differential diagnosis between ALCL and cHL can be made by expression of cytotoxic molecules such as Granzyme B, Perforin and T-cell-restricted intracellular antigen-1 (TIA1), EMA and CD45/LCA which are typical of ALCL, while positivity for CD15 (in 70% of cases), PAX5 (90%) and LMP1 (in 50%) are typical of cHL. To distinguish ALCL from ALK-1-positive large B cell lymphomas the lack of CD30 expression is critical and the t(2;5) translocation cannot be demonstrated in the B cell lymphoma [19].

We conclude that this case represents a very rare manifestation of an ALCL in soft tissue. Despite the anaplastic appearance a favorable outcome was possible after promptly applying aggressive chemotherapy. The expression of the NMP-ALK-1 protein and the young age of the patient might have influenced the positive outcome.

## Consent

Written informed consent was obtained from the patient, respectively the guardians for publication of this case report and accompanying images. A copy of the written consent is available for review to the Editor-in-Chief of this journal.

## Competing interests

The authors declare that they have no competing interests.

## Authors' contributions

TG and AM drafted the manuscript and analysed the data. EG and AR served as reference pathologists. TS, HPS and MD were leadingly involved into the clinical examination and the treatment of the patient. DD carried out MRI and subsequent imaging analyses. All authors read and approved the manuscript.
